# Clinical and Genetic Characteristics of Mitochondrial Encephalopathy Due to *FOXRED1* Mutations: Two Chinese Case Reports and a Review of the Literature

**DOI:** 10.3389/fneur.2021.633397

**Published:** 2021-02-03

**Authors:** Chaoping Hu, Qiong Xu, Jin Shen, Yi Wang

**Affiliations:** ^1^Department of Neurology, Children's Hospital of Fudan University, Shanghai, China; ^2^Department of Child Health Care, Children's Hospital of Fudan University, Shanghai, China; ^3^Department of Radiology, Children's Hospital of Fudan University, Shanghai, China

**Keywords:** FOXRED1, mitochondrial encephalopathy, complex I defect, neuroimage, cystic encephalomalacia

## Abstract

**Background:** As one of the assembly factors of complex I in the mitochondrial respiratory chain, FOXRED1 plays an important role in mitochondrial function. However, only a few patients with mitochondrial encephalopathy due to FOXRED1 defects have been reported.

**Methods:** Two Chinese patients with mitochondrial encephalopathy due to mutations in *FOXRED1* were identified through trio whole-exome sequencing. The clinical presentation, laboratory data, brain imaging findings, and genetic results were collected and reviewed. All previously reported cases with *FOXRED1*-related mitochondrial encephalopathy were collected using a PubMed search, and their data were reviewed.

**Results:** Two patients presented with severe neurodevelopmental delay, epilepsy, high lactic acid levels, and remarkable diffuse brain atrophy and polycystic encephalomalacia during early infancy. Trio whole-exome sequencing revealed compound heterozygous variants in both patients: one case harbored a c.606_607delAG frameshift variant and a c.1054C>T (p.R352W) variant. At the same time, the other carried a novel c.352C>T (p.Q118X) variant and a reported c.1054C>T (p.R352W) variant. To date, nine patients have been reported with *FOXRED1* defects, including our two cases. The most common presentations were neurodevelopment delay (100%), epilepsy (80%), poor feeding (30%), and vision loss (20%). Multisystem involvement comprised cardiovascular dysfunction (30%), abnormal liver function (20%), and hypoglycemia (10%). The neuroimaging results ranged from normal to severe cerebral atrophy and polycystic encephalomalacia in early infancy. Eleven pathogenic variants in FOXRED1 have been reported, comprising six missense variants, two non-sense variants, two frameshift variants, and one splice variant; among these the c.1054C>T (p.R352W) and c.612_615dupAGTG (p.A206SfsX15) variants are more common.

**Conclusion:** *FOXRED1*-related mitochondrial disorders have high clinical and genetic heterogeneity. Our study expanded the clinical and genetic spectrum of *FOXRED1* defects. Early infantile onset and progressive encephalopathy are the most common clinical presentations, while the variants c.1054C>T (p.R352W) and c.612_615dupAGTG (p.A206SfsX15) may be critical founder mutations.

## Introduction

Mitochondrial diseases (MDs) are by far the largest class of inborn errors of metabolism, with a collective incidence of 1~1.6 in 5,000 (1, 2), among which defects in complex I (CI) activity are the most common (3). Complex I comprises seven subunits encoded by mitochondrial DNA (mtDNA) and 38 subunits encoded by nuclear DNA (nDNA), and mutations of these genes account for 25 and 20% of CI deficiency, respectively (4). The remaining half of CI deficiency is believed to be caused by mutations in ancillary factors necessary for proper CI assembly and functioning (5). To date, 15 assembly factors have been identified (6).

FAD-dependent oxidoreductase domain-containing protein 1 (FOXRED1) encodes a FAD-dependent oxidoreductase complex-I-specific molecular chaperone (5), which has recently been identified to play an important role in the mid-late stages of CI assembly (7). By 2019, only seven patients with mitochondrial CI deficiency caused by mutations in the *FOXRED1* gene had been reported (5, 8–12). Here, we describe two Chinese patients with compound heterozygous variants in the *FOXRED1* gene, both presenting with neonatal onset severe progressive mitochondrial encephalopathy and characteristic neuroimaging of early cerebral atrophy and encephalomalacia. In this study, we also reviewed all the data of previously reported patients with mitochondrial encephalopathy caused by mutations in the *FOXRED1* gene and summarized the genetic and clinical findings of *FOXRED1*-related MDs.

## Patients and Methods

### Patients

Two patients with suspected mitochondrial encephalopathy due to *FOXRED1* variants were enrolled from the Department of Neurology, Children's Hospital of Fudan University, between September 2019 and November 2020. The clinical presentation, laboratory data, brain imaging findings, and genetic results were collected and reviewed.

Ethical approval for the study was obtained from the health authority ethical committee of Children's Hospital of Fudan University. All the blood samples were collected after obtaining written consent from the parents of each patient in compliance with the Declaration of Helsinki.

### DNA Isolation, Molecular Tests, and Analysis

Both patients received trio whole-exome sequencing tests, which were performed at the Translational Research Center of Children's Hospital of Fudan University. Nuclear DNA was extracted from 2-mL aliquots of whole-blood leukocytes obtained from the patients and their parents. NGS of nDNA was performed using genomic DNA from each patient on a HiSeq 2000 platform (Illumina). After mapping to the reference human genome (UCSC hg 19), the sequencing data were sorted and merged, with duplicate reads removed from BAM files using SAM tools version 0.1.16. Sanger sequencing was performed to validate the variations and determine their parental origin.

The variants were classified as “pathogenic” or “likely pathogenic” according to the variant interpretation guidelines of the American College of Medical Genetics and Genomics (13).

### MR Techniques and Protocols

Brain MRI of both patients was conducted at the Department of Radiology, Children's Hospital of Fudan University, and included high-resolution spin-echo T1-weighted images, spin-echo T2-weighted images, T2-weighted images with suppression of the cerebrospinal fluid (CSF) signal [fluid-attenuated inversion recovery (FLAIR)], and diffusion-weighted images sensitive to water diffusivity.

### Literature Review

Previously reported cases with *FOXRED1*-related mitochondrial encephalopathy were identified through a PubMed search. The clinical, biochemical, neuroimaging, molecular data and neurodevelopmental outcomes were obtained from the respective references, reviewed and compared with those of our present cases.

## Results

### Case Description

**Figure 1:**
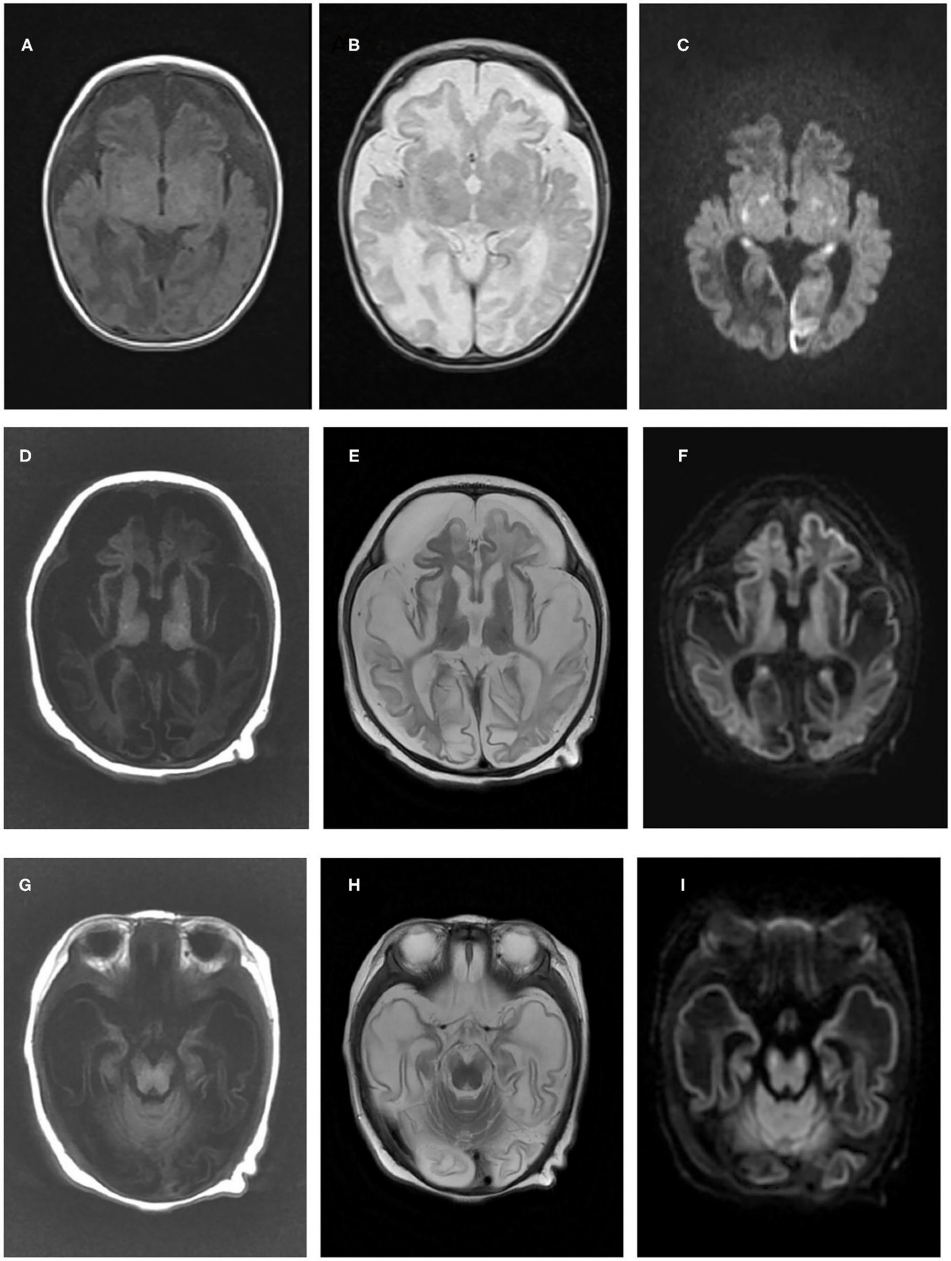
Brain magnetic resonance imaging results of the two patients in the present study. Brain magnetic resonance imaging of patient one in our study at 1 month old revealed cerebral atrophy and partial encephalomalacia, a diffuse abnormal signal in the cerebral white matter, with a low signal in T1WI **(A)** and a high signal in T2WI **(B)**, and high signals in the bilateral occipital and temporal lobes, basal ganglia and thalamus **(C)**. An MRI of patient two at 3 months old indicated polycystic encephalomalacia and diffuse cerebral atrophy, with diffuse abnormal signals in the brain, low signals on T1WI and high signals on T2WI and DWI **(D–F)**, and a high signal in the cerebral peduncle **(G–I)**.

Case 1 is a 10-month-old boy, the first child of healthy nonconsanguineous Chinese parents of Han nationality, who had been mentioned in our previous report (14). During late pregnancy, a small head circumference was noticed. He was born at term in an uneventful delivery, with a birth weight of 3.35 kg. Three weeks after birth, he was admitted to the NICU at our hospital because of difficulty in feeding, little voluntary movement, and unsatisfactory weight gain. Physical examination showed a poor response, microcephaly, high muscle tone, muscle weakness, and absent primary reflection. The laboratory investigation revealed an elevated lactic acid level of 5.0 mmol/L (normal range: <2.1 mmol/L). The results of liver function, blood ammonia, glucose, creatine kinase, thyroid function, and intrauterine infection investigation (TORCH) were unremarkable. Echocardiography showed patent ductus arteriosus (PDA) and pulmonary artery hypertension. Brain MRI at 1 month revealed profound diffuse abnormal signals, brain atrophy, and partial encephalomalacia (Figures 1A–C). Neonatal infection and encephalopathy were diagnosed. Following symptomatic treatment with anti-infection and nutrition guidance, 7 days later, he was discharged because of improvement in mental state and feeding.

At ~1 month of age, partial tonic and myoclonic epilepsy occurred refractory to levetiracetam, sodium valproate, and topiramate. Furthermore, the patient developed poor tracking gradually. Sleep EEG showed multispike slow waves in the bilateral temporal region (predominantly left). Visual evoked potentials showed poor waveform differentiation. The blood tandem mass spectrometry results were normal, while urine gas mass spectrometry indicated elevated 4-hydroxyphenylacetic acid and aconitrate levels.

At 10 months old, the patient was admitted to our intensive care unit again for dysphagia and difficult breathing. He was intubated with ventilator support and nasal feeding, and other symptomatic treatments were introduced, including acidosis correction and anti-infection medication. 5 days later, his parents took him home, and he failed to survive.

Trio whole-exome sequencing performed when the patient was 10 months old revealed compound heterozygous variants in the *FOXRED1* gene (NM_017547), including a frameshift c.606_607delAG variant in exon 5, which has been submitted to the public database ClinVar (http://www.ncbi.nlm.nih.gov/clinvar, submission number: SUB6664717) and a c.1054C>T (p.R352W) missense variant in exon 9. The father was heterozygous for c.606_607delAG, while the mother was heterozygous for c.1054C>T (p.R352W). The patient's c.1054C>T (p.R352W) variant has never been reported in the 1,000 Genomes Project, and only three individuals who were heterozygous for c.1054C>T (p.R352W) were reported in the ExAC database. Complex I deficiency was identified in one patient with homozygous variants of c.1054C>T (p.R352W) (5). According to the ACMG criteria 2015 guidelines (13), it is classified as likely pathogenic (PM1, PM2, PP3, PP5). The c.606_607delAG variant causes a frameshift change, which interrupts the transcription of subsequent sequences. It has never been reported in the 1,000 Genomes Project or ExAC database, and it is considered pathogenic (PVS1, PS4, PM2, PP1) according to the ACMG 2015 guideline (13), as described in our previous report (14).

Case 2 is a 3-month-old boy, the first child of healthy non-consanguineous parents of Han ethnicity. His mother had a history of preeclampsia during late pregnancy, and he was born by cesarean section at 36^+6^ weeks because of abnormal fetal heart monitoring, with a normal Apgar score. After birth, less movement of the left limbs was noticed without any interference. At 3 months, he was taken to our neurology clinic because of developmental delay, poor tracking and response, and a cluster of limb and head shaking. Physical examination showed no remarkable neurological signs. Laboratory investigation showed slight elevation in liver function (ALT 51 IU/L, normal range: 9–50 IU/L; AST 60 IU/L, normal range: 15–40 IU/L), lactic acid (Lac 4.5–6.2 mmol/L, Nor: <2.1 mmol/L), and creatine kinase (CK 337 IU/L, normal range: 0–164 IU/L). Intrauterine infections, such as CMV and HSV, were expelled. Blood ammonia, thyroid function, and blood serial mass spectrum were normal, while the urine mass spectrum test indicated slightly elevated 2-ketoglutarate and aconitrate levels. Electroencephalography (EEG) was performed and revealed slow waves (2–3 Hz) and low voltage in the background without epileptic discharge. Brain MRI at 3.5 months showed impressive diffused abnormal signals in the cortex and white matter, polycystic encephalomalacia, cerebral atrophy, and bilateral subdural effusion (Figures 1D–I). Neonatal encephalopathy and epilepsy were diagnosed, and clonazepam was prescribed. However, no obvious improvement was observed until now.

Additionally, trio whole-exome sequencing was conducted and revealed compound heterozygous variants in the *FOXRED1* gene (NM_017547), including a novel c.352C>T (p.Q118X) nonsense variant in exon 3, which has been submitted to the public database ClinVar (http://www.ncbi.nlm.nih.gov/clinvar, submission number: SUB 8878161) and a reported c.1054C>T (p.R352W) missense variant in exon 9. The father was heterozygous for c.352C>T (p.Q118X), while the mother was heterozygous for c.1054C>T (p.R352W). The variant c.352C>T (p.Q118X), which has never been reported in the 1000 Genomes Project or ExAC database, leads to premature termination of translation and causes protein shortening; according to the ACMG 2015 guideline (13), it is pathogenic (PVS1, PM2, PP3).

### Literature Review of Previously Reported Patients

**Figure 2:**
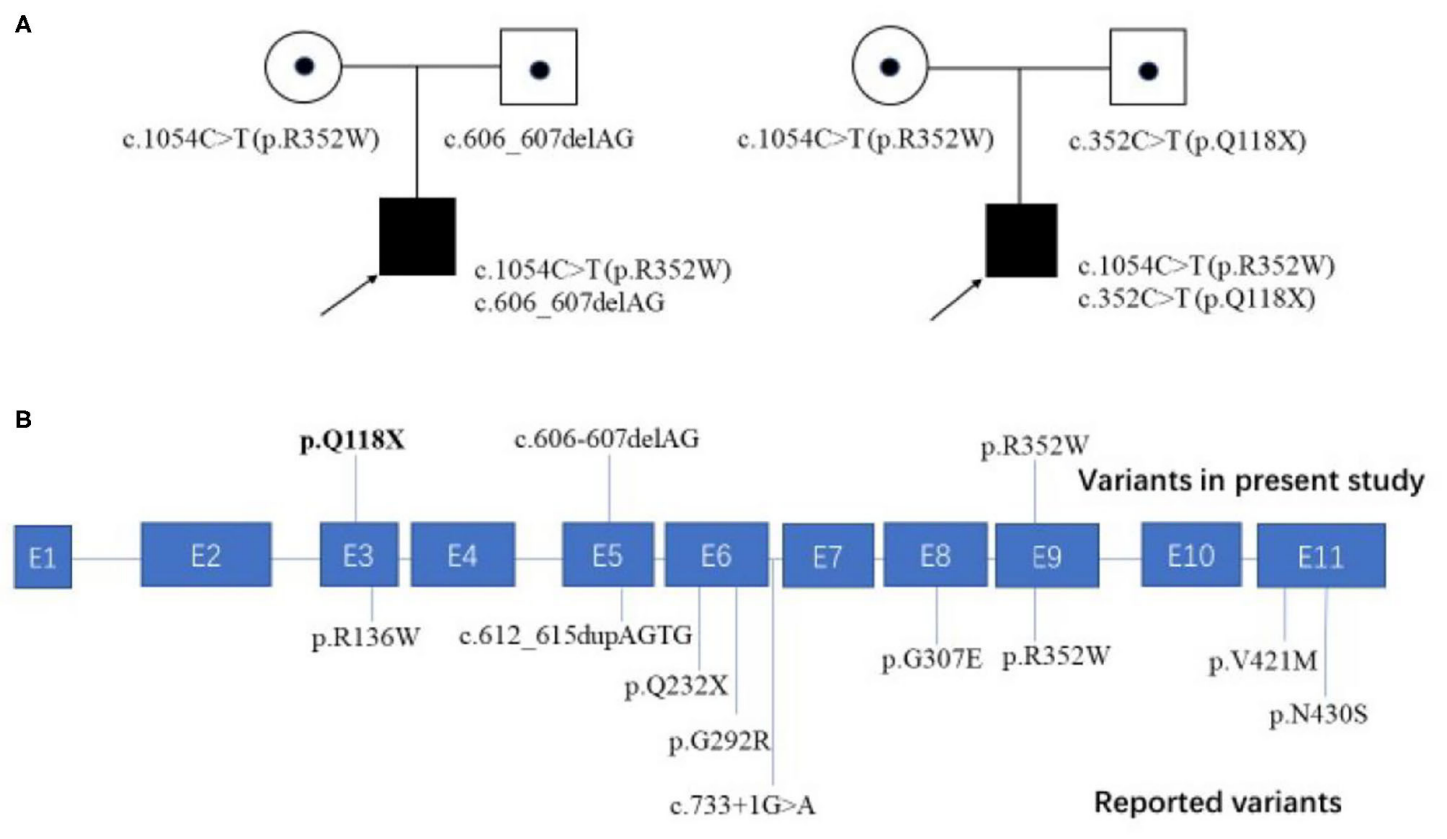
Family pedigrees of our cases and all the reported pathogenic variants mapped on the *FOXRED1* gene structure. **(A)** Two patients from unrelated families in our study both harbored compound heterozygous mutations in the *FOXRED1* gene. **(B)** The *FOXRED1* gene comprises 11 exons, which are illustrated in blue. The pathogenic variants identified in this study are illustrated on the upper side of the exons with novel variants highlighted in bold; other previously reported variants are on the lower side.

To date, nine patients with *FOXRED1-*related mitochondrial encephalopathy from 8 unrelated families have been reported, including our two cases (Table 1). All the patients had an early onset except for one patient who had a learning disorder and clumsiness at 4 years old (Table 1, case 8). Developmental delay or the loss of acquired milestones was most common in these patients, followed by epilepsy and vision impairment. Among seven patients with neuroimaging results, five revealed diffuse abnormal signals in the cortex and deep white matrix during early infancy. Eleven variants were reported in the *FOXRED1* gene, comprising six missense variants, two non-sense variants, two frameshift variants, and one splicing variant (Figure 2).

**Table 1:** Clinical and genetic characteristics of FOXRED1-related mitochondrial encephalopathy.

**Case**	**FOXRED1 variants**		**Prenatal period**	**Onset**	**CNS symptoms**				**Multisystem presentation**	**Brain MRI**	**Lac (<2.1 mmol/L)**	**Survival**
	**Variants**	**Zygo**			**Developmental delay**	**Epilepsy**	**Vision**	**Other**				
1 (8)	c.694C>T (p.Q232X) c.1289A>G (p.N430S)	Het	–	neonatal	loss of milestones; no language	+	N/A	hypotonia	hypoglycemia	decreased attenuation in the putamen bilaterally; significant cerebellar atrophy (6y)	high	alive (22 y)
2 (5)	c.1054C>T (p.R352W)	Hom	–	neonatal	severe	myoclonic	roving eyes bilateral optic atrophy	poor feeding	mild non-obstructive left ventricular hypertrophy; hepatomegaly	delayed myelination, ventricular dilatation and abnormal signal in the thalami and basal ganglia (8 m)	6.8	alive (10 y)
3 (9)	c.1308G>A (p.V421M)	Hom	–	infantile	+	+	N/A	N/A	N/A	N/A	N/A	N/A
4 (10)	c.406C>T (p.R136W) c.612_615dupAGTG (p.A206SfsX15)	Het	–	<6 m	+	–	–	–	–	N/A	–	progressive
5 (11)	c.612_615dupAGTG (p.A206SfsX15) c.874G>A (p.G292R)	Het	IUGR	neonatal	+	generalized	N/A	mild hypertonia, wrist and ankle contracture	pulmonary artery hypertension, enlargement of the right atrium/ventricle, patent ductus arteriosus; patent foramen ovale	diffusely abnormal brain with large periventricular cysts, edematous white matter, delayed myelination, thin and undersulcated cortex particularly anteriorly (13 d)	20	died at 3 m
6 (12)	c.920G>A (p.G307E) c.733+1G>A	Het	IUGR	2 m	+	refractory	latent strabismus of the right eye	ataxia	Bronchospasm episodes during infancy	normal (2 m, 4 y, 7 y 3 m)	elevated	alive (15 y)
7 (12)	c.920G>A (p.G307E) c.733+1G>A	Het	–	4 y	learning disorders	–		clumsiness	–	normal	elevated after exercise	alive (19 y)
8 (14) (this study)	c.606_607delAG c.1054C>T (p.R352W)	Het	IUGR	neonatal	severe	partial tonic; myoclonic; refractory	vision loss	poor feeding	patent ductus arteriosus; patent foramen ovale; pulmonary artery hypertension	diffuse abnormal signal in the cerebral white matter, bilateral occipital and temporal lobe, basal ganglia and thalamus, cerebral atrophy, partial encephalomalacia (1 m)	5.0	died at 3 y
9(this study)	c.352C>T (p.Q118X) c.1054C>T (p.R352W)	Het	preeclampsia, preterm at 36^+6^w	neonatal	severe	myoclonic	–	–	elevated liver function	polycystic encephalomalacia, diffuse cerebral atrophy, diffuse abnormal signal in the brain, agenesis of corpus collosum (3 m)	6.2	progressive (10 m)

*DD, developmental delay; zygo, heterozygous or homozygous state; IUGR, intrauterine growth restriction; –, normal or within normal range; N/A, information not available. Cases 6 and 7 are siblings*.

## Discussion

FOXRED1, a mitochondria-targeted 486-amino acid FAD-dependent oxidoreductase (15), is one of the 15 assembly factors of complex I, the first and largest complex of the OXPHOS system. Mutations in *FOXRED1* have been reported to be associated with Leigh syndrome (7, 8) and infantile-onset mitochondrial encephalopathy (5). Here, we described two Chinese patients with neonatal-onset progressive mitochondrial encephalopathy with a new neuroimaging pattern caused by compound heterozygous variants in the *FOXRED1* gene, including one novel nonsense c.352C>T (p.Q118X) variant, which expanded the clinical and genetic spectrum of *FOXRED1*-related mitochondrial disorders.

Like other mitochondrial disorders, there is high clinical heterogeneity in *FOXRED1*-related mitochondrial disorders (Table 1), which range from mild learning disorders and clumsiness (12) to severe progressive mitochondrial encephalopathy and even failure to survive (11). The most common symptoms comprise developmental delay (100%), epilepsy (80%), vision loss (20%), and dysphagia (20%). Multisystem involvement, such as cardiovascular dysfunction, abnormal liver function, and hypoglycemia, has also been observed. In our study, we reported two patients with neonatal onset, severe progressive encephalopathy with lactic acidosis, and characteristic polycystic encephalomalacia on brain MRI. Among these *FOXRED1*-mutated patients, eight patients had an onset in infancy (8/9, 88.9%), and five patients had an even earlier neonatal onset (5/9, 55.6%); thus, the percentage of both is much higher than that (~30%) of infantile-onset or neonatal-onset cases among overall nuclear-encoded complex I defects (16). Among the infantile-onset patients, three cases had IUGR (3/8, 37.5%). Additionally, among the five cases with abnormal brain MRI results (5/7, 71.4%), four showed remarkable lesions during infancy (ranging from 13 days after birth to 8 months). These phenomena indicate early damage in the brains of *FOXRED1*-mutated patients. Studies in different mammalian species have shown that FOXRED1 expression increases rapidly during embryonic development (17), and proinflammatory conditions during pregnancy, such as IUGR and preeclampsia, can dramatically increase ROS production, placing even higher demands on the oxidative stress defense systems (18). Accordingly, in 2015, one researcher hypothesized that, as a sarcosine oxidase, the catalytic activity of FOXRED1 protects the developing fetus from oxidative stress during pregnancy (19), a notion that is consistent with our investigations. However, more evidence must be elucidated.

All mitochondrial encephalopathy patients with *FOXRED1* mutations are inherited in an autosomal recessive pattern. To date, 11 pathogenic variants in the *FOXRED1* gene have been reported (Figure 2), including six missense variants, two non-sense variants, two frameshift variants, and one splice variant. Among these variants, c.1054C>T (p.R352W) and c.612_615dupAGTG (p.A206SfsX15) were more common than others. Concerning c.1054C>T (p.R352W), the transition from a highly conserved arginine residue to the much larger tryptophan possibly results in the tryptophan sidechain being closer to the FAD-binding site, thus interfering with FAD binding. This variant was found in two Chinese patients (cases 8 and 9) and one Iranian-Jewish patient (case 2). According to public databases such as ExAC, c.1054C>T (p.R352W) has a frequency of 0.01% in East Asians (much higher than in other populations); thus, it may be a hot spot in East Asian *FOXRED1*-mutated MDs and may play a critical role in molecular screening in Asian patients. The duplication c.612_615dupAGTG (p.A206SfsX15) variant, which changes alanine 206 to a serine residue, causes a frameshift and creates a premature stop codon at position 15 of the new reading frame, leading to a loss of normal protein function either through protein truncation or nonsense-mediated mRNA decay and is predicted to be pathogenic (11). The c.612_615dupAGTG (p.A206SfsX15) variant has a relatively high frequency of 0.08% in Ashkenazi Jews; however, the ethnic group of two patients who were reported to carry the variant was unavailable. Further investigation is needed to evaluate the hot spots.

Loss of FOXRED1 function mainly leads to a reduction in the amount of fully assembled complex I (5). In 2017, the characterization of fibroblast lines from *FOXRED1*-mutated patients demonstrated that FOXRED1 is involved in the middle to late stages of complex I assembly (7). However, another study demonstrated that FOXRED1 function likely involves the assembly of two flavoprotein-containing OXPHOS complexes (Complexes I and II) and is cell-type specific; for example, FOXRED1 is required for complex II assembly in myoblasts (9). However, the precise molecular role of FOXRED1 in the OXPHOS system and pregnancy remains to be elucidated.

No genotype-phenotype correlation seems to exist for *FOXRED1*-related mitochondrial encephalopathy, similar to many other mitochondrial disorders. No curative methods are currently available for *FOXRED-1*-related complex I deficiency patients. With palliative care, two patients failed to survive, and most of the living patients showed a progressing disease course, severe disability and poor quality of life.

## Conclusion

In this study, we reported two Chinese patients with neonatal-onset severe progressive encephalopathy with lactic acidosis and characteristic polycystic encephalomalacia on brain MRI, expanding the clinical and molecular genetic spectrum of *FOXRED1*-related mitochondrial encephalopathy. Based on the literature review, early infantile onset and progressive encephalopathy are the most common clinical presentations, always with high mortality and disability. Among the mutations, c.1054C>T (p.R352W) and c.612_615dupAGTG (p.A206SfsX15) are more common, which may be helpful in molecular genetic analysis.

## Data Availability Statement

The datasets generated for this study can be found in online repositories. The names of the repository/repositories and accession number(s) can be found below: ClinVar (http://www.ncbi.nlm.nih.gov/clinvar, submission number: SUB6664717 and SUB8878161).

## Ethics Statement

The studies involving human participants were reviewed and approved by The health authority ethical committee of Children's Hospital of Fudan University. Written informed consent to participate in this study was provided by the participants' legal guardian/next of kin. Written informed consent was obtained from the minor(s)' legal guardian/next of kin for the publication of any potentially identifiable images or data included in this article.

## Author Contributions

CH prepared and drafted this manuscript. JS conducted the brain MRI tests. YW and QX fulfill data analysis and approve for the submission. All authors contributed to the article and approved the submitted version.

## Conflict of Interest

The authors declare that the research was conducted in the absence of any commercial or financial relationships that could be construed as a potential conflict of interest.

## References

[1] Stenton SL Prokisch H. Genetics of mitochondrial diseases: identifying mutations to help diagnosis. EBioMed. (2020) 56:102784. 10.1016/j.ebiom.2020.102784 32454403 PMC7248429

[2] Skladal D Halliday J Thorburn DR. Minimum birth prevalence of mitochondrial respiratory chain disorders in children. Brain. (2003) 126:1905–12. 10.1093/brain/awg170 12805096

[3] Fiedorczuk K Sazanov LA. Mammalian mitochondrial Complex I structure and disease-causing mutations. Trends Cell Biol. (2018) 28:835–67. 10.1016/j.tcb.2018.06.006 30055843

[4] Thorburn DR Sugiana C Salemi R Kirby DM Worgan L Ohtake A. Biochemical and molecular diagnosis of mitochondrial respiratory chain disorders. Biochim Biophys Acta. (2004) 1659:121–8. 10.1016/j.bbabio.2004.08.006 15576043

[5] Fassone E Duncan AJ Taanman JW Pagnamenta AT Sadowski MI Holand T. FOXRED1, encoding an FAD-dependent oxidoreductase complex-I-specific molecular chaperone, is mutated in infantile-onset mitochondrial encephalopathy. Hum Mol Genet. (2010) 19:4837–47. 10.1093/hmg/ddq414 20858599 PMC4560042

[6] Formosa LE Dibley MG Stroud DA Ryan MT. Building a complex complex: Assembly of mitochondrial respiratory chain complex I. Semin Cell Dev Biol. (2018) 76:154–62. 10.1016/j.semcdb.2017.08.011 28797839

[7] Formosa LE Mimaki M Frazier AE McKenzie M Stait TL Thorburn DR. Characterization of mitochondrial FOXRED1 in the assembly of respiratory chain complex I. Hum Mol Genet. (2015) 24:2952–65. 10.1093/hmg/ddv058 25678554

[8] Calvo SE Tucker EJ Compton AG Kirby DM Crawford G Burtt NP. High-throughput, pooled sequencing identifies mutations in NUBPL and FOXRED1 in human complex I deficiency. Nat Genet. (2010) 42:851–8. 10.1038/ng.659 20818383 PMC2977978

[9] Zurita Rendon O Antonicka H Horvath R Shoubridge EA. A mutation in the Flavin adenine dinucleotide-dependent oxidoreductase FOXRED1 results in cell-type-specific assembly defects in oxidative phosphorylation Complexes I and II. Mol Cell Biol. (2016) 36:2132–40. 10.1128/MCB.00066-16 27215383 PMC4968213

[10] Haack TB Madignier F Herzer M Lamantea E Danhauser K Invernizzi F. Mutation screening of 75 candidate genes in 152 complex I deficiency cases identifies pathogenic variants in 16 genes including NDUFB9. J Med Genet. (2012) 49:83–9. 10.1136/jmedgenet-2011-100577 22200994

[11] Apatean D Rakic B Brunel-Guitton C Hendson G Bai R Sargent MA. Congenital lactic acidosis, cerebral cysts and pulmonary hypertension in an infant with FOXRED1 related complex I deficiency. Mol Genet Metab Rep. (2019) 18:32–8. 10.1016/j.ymgmr.2018.12.006 30723688 PMC6349952

[12] Barbosa-Gouveia S Gonzalez-Vioque E Borges F Gutierrez-Solana L Wintjes L Kappen A. Identification and characterization of new variants in FOXRED1 gene expands the clinical spectrum associated with mitochondrial Complex I deficiency. J Clin Med. (2019) 8:1262. 10.3390/jcm8081262 31434271 PMC6723710

[13] Richards S Aziz N Bale S Bick D Das S Gastier-Foster J. Standards and guidelines for the interpretation of sequence variants: a joint consensus recommendation of the American College of Medical Genetics and Genomics and the Association for Molecular Pathology. Genet Med. (2015) 17:405–24. 10.1038/gim.2015.30 25741868 PMC4544753

[14] Hu C Li X Zhao L Shi Y Wu B Zhou S. Clinical and molecular characterization of pediatric mitochondrial disorders in southof China. Eur J Med Genet. (2020) 63:103898. 10.1016/j.ejmg.2020.103898 32348839

[15] Lemire BD. Evolution of FOXRED1, an FAD-dependent oxidoreductase necessary for NADH:ubiquinone oxidoreductase (Complex I) assembly. Biochim Biophys Acta. (2015) 1847:451–7. 10.1016/j.bbabio.2015.01.014 25681241

[16] Fassone E Rahman S. Complex I deficiency: clinical features, biochemistry and molecular genetics. J Med Genet. (2012) 49:578–90. 10.1136/jmedgenet-2012-101159 22972949

[17] Xie D Chen CC Ptaszek LM Xiao S Cao X Fang F. Rewirable gene regulatory networks in the preimplantation embryonic development of three mammalian species. Genome Res. (2010) 20:804–15. 10.1101/gr.100594.109 20219939 PMC2877577

[18] Webster RP Roberts VH Myatt L. Protein nitration in placenta - functional significance. Placenta. (2008) 29:985–94. 10.1016/j.placenta.2008.09.003 18851882 PMC2630542

[19] Lemire BD. Glutathione metabolism links FOXRED1 to NADH:ubiquinone oxidoreductase (complex I) deficiency: a hypothesis. Mitochondrion. (2015) 24:105–12. 10.1016/j.mito.2015.07.009 26235939

